# Supplementary material for: Mechanisms governing the pioneering and redistribution capabilities of the non-classical pioneer PU.1
Source: Nat Commun. 2020 Jan 21;11:402. doi: 10.1038/s41467-019-13960-2 (PMC6972792; doi:10.1038/s41467-019-13960-2)
Supplement: Supplementary file 7 — Source data [file 41467_2019_13960_MOESM7_ESM.zip › Source_Data/FigureS5/FigureS5A_MotifsCommonPeaks/homerResults/motif14.similar.html]

motif14

## Information for motif14

A
C
T
G
A
G
T
C
C
G
A
T
A
C
G
T
A
T
C
G
A
G
T
C
A
C
T
G
  
Reverse Opposite:  

A
G
T
C
A
C
T
G
A
T
G
C
C
G
T
A
C
G
T
A
A
C
T
G
A
G
T
C
  

|  |  |
| --- | --- |
| p-value: | 1e-113 |
| log p-value: | -2.610e+02 |
| Information Content per bp: | 1.948 |
| Number of Target Sequences with motif | 1007.0 |
| Percentage of Target Sequences with motif | 11.33% |
| Number of Background Sequences with motif | 1724.9 |
| Percentage of Background Sequences with motif | 4.54% |
| Average Position of motif in Targets | 98.4 +/- 56.1bp |
| Average Position of motif in Background | 96.4 +/- 70.8bp |
| Strand Bias (log2 ratio + to - strand density) | -0.0 |
| Multiplicity (# of sites on avg that occur together) | 1.07 |
| Motif File: | file (matrix) reverse opposite |

### Similar de novo motifs found

|  |  |  |  |  |  |  |  |
| --- | --- | --- | --- | --- | --- | --- | --- |
| Rank | Match Score | Redundant Motif | P-value | log P-value | % of Targets | % of Background | Motif file |
| 1 | 0.804 | G T A C A C T G A T G C G C T A C G A T T C A G T G A C A C T G | 1e-62 | -144.802202 | 6.28% | 2.48% | motif file (matrix) |
| 2 | 0.765 | T A C G A G C T C T A G T G A C T C A G A G T C T G C A C A G T A C T G A G T C A C T G G A T C T G C A | 1e-25 | -58.012967 | 2.16% | 0.78% | motif file (matrix) |
| 3 | 0.765 | T A C G A C G T C T A G T A G C C T A G A G T C T G C A C A G T A C T G A G T C A C T G G A T C | 1e-23 | -54.795677 | 2.28% | 0.89% | motif file (matrix) |
| 4 | 0.763 | C A G T T C A G G T A C T C A G A G T C T G C A C G A T A C T G A G T C A T C G | 1e-23 | -54.614576 | 2.52% | 1.04% | motif file (matrix) |
| 5 | 0.758 | A G C T C T A G T G A C T C A G G T A C G T C A C A G T A T C G A G T C A C T G G A T C | 1e-23 | -53.983714 | 3.00% | 1.36% | motif file (matrix) |
| 6 | 0.788 | C T A G G T A C T C A G A G T C T G C A C A G T A C T G A G T C A C T G | 1e-18 | -43.512937 | 2.60% | 1.22% | motif file (matrix) |
